# DIFFERENT SURGICAL METHODS IN COLON ANASTOMOSIS: EXPERIMENTAL STUDY

**DOI:** 10.1590/0102-672020210002e1662

**Published:** 2022-06-24

**Authors:** Hasan CANTAY, Ugur AYDIN, Isa OZAYDIN, Turgut ANUK, Serap Koral TASCI, Ugur YILDIZ, Dilem ERMUTLU, Ozgur AKSOY

**Affiliations:** 1 Kafkas University, Faculty of Medicine, Department of General Surgery, TR-36000 Kars, TURKEY;; 2 Kafkas University, Faculty of Veterinary Medicine, Department of Surgery, TR-36000 Kars, TURKEY;; 3 Kafkas University, Faculty of Veterinary Medicine, Department of Histology and Embryology, TR-36000 Kars, TURKEY.

**Keywords:** Colon, Surgical Mesh, Omentum, Wistar, Rats, Anastomosis, Surgical, Colo, Telas Cirúrgicas, Omento, Ratos Wistar, Anastomose Cirúrgica

## Abstract

Although many methods have been defined for colonic anastomosis, anastomotic leak still remains important for sepsis control and successful healing.

**AIM:**

The purpose of this study was to compare the effects of conventional suture, polyglactin 910 mesh, and omental flap coverage on healing and anastomotic leak in experimental colonic anastomosis in rats.

**METHOD:**

This study was conducted on 18 Wistar rats and the animals were divided into three groups as follows: Group 1: primary suture group; Group 2: primary suture *plus* polyglactin 910 mesh group; and Group 3: primary suture *plus* omental flap coverage group. Groups were compared in terms of anastomotic bursting pressure, inflammation, fibroblastic activity, neovascularization, and collagen amount.

**RESULTS:**

There was a statistically significant difference in anastomotic bursting pressure between Groups 1 and 2 and between Groups 1 and 3 (p=0.004, p<0.05). There was a significant difference in fibroblastic activity between Groups 1 and 3 (p=0.011, p<0.05) and between Groups 2 and 3 (p=0.030, p<0.05). There was a significant difference in neovascularization and collagen between Groups 1 and 2 and between Groups 1 and 3 (p<0.05).

**CONCLUSION:**

This experimental study found that polyglactin 910 mesh and omental flap coverage for colocolic anastomoses improved the physical strength and healing of the anastomosis compared to conventional hand-stitched anastomoses. The polyglactin may be a safe alternative to 910 mesh in cases where the omental flap coverage cannot be used in the colonic anastomosis.

## RESUMO - RACIONAL

Embora muitos métodos tenham sido definidos para anastomose colônica, a fistula anastomótica ainda permanece importante para o controle da sepse e a cura bem-sucedida.

**OBJETIVO:**

comparar os efeitos da sutura convencional, tela de poliglactina 910 e cobertura de retalho omental na cicatrização e extravasamento anastomótico em anastomose colônica experimental em ratos.

**MÉTODO:**

estudo realizado em 18 ratos Wistar, sendo os animais divididos em 3 grupos. Grupo 1: Grupo de sutura primária; Grupo 2: sutura primária com malha de poliglactina 910; Grupo 3: Grupo sutura primária com cobertura de retalho omental. Os grupos foram comparados em termos de pressão de ruptura anastomótica, inflamação, atividade fibroblástica, neovascularização e quantidade de colágeno.

**RESULTADOS:**

houve diferença estatisticamente significativa na pressão de ruptura da anastomose entre os Grupos 1 e 2 e os Grupos 1 e 3 (p=0,004, p<0.05). Houve uma diferença significativa na atividade fibroblástica entre os Grupos 1 e 3 (p=0,011, p<0.05) e os Grupos 2 e 3 (p=0,030, p<0.05). Houve uma diferença significativa na neovascularização e colágeno entre os Grupos 1 e 2 e entre os Grupos 1 e 3 (p<0,05, p<0.05).

**CONCLUSÃO:**

o estudo experimental demonstrou que a tela de poliglactina 910 e a cobertura do retalho omental para anastomoses colocólicas melhoraram a resistência física e a cicatrização da anastomose em comparação com as anastomoses suturadas manualmente convencionais. A poliglactina pode ser uma alternativa segura à tela 910 nos casos em que a cobertura do retalho omental não pode ser utilizada na anastomose colônica.

## INTRODUCTION

Although colorectal surgery is not a difficult procedure in practice, there is a risk of complications such as fistula, bleeding, anastomotic stenosis, and anastomotic leak. One of the most common complications is anastomotic leak, which leads to high morbidity and mortality rates. Therefore, anastomotic leak has been one of the most investigated topics in colorectal surgery^11^ ^,^ ^24^. In the literature, there are experimental studies on anastomotic healing which involve methods such as reducing the number of sutures, dexpanthenol, coenzyme Q10, krill oil, and fish oil^3^ ^,^ ^6^ ^,^ ^9^ ^,^ ^17^. The indication for surgery and many factors related to the operated patient is also as effective as the technique used in colorectal surgery. Therefore, when planning the surgical procedure, considering that many factors, such as the level of the anastomosis and the environment where the anastomosis will be performed, will have an effect on the outcome, it is important to decide which technique or techniques will achieve the best result instead of determining which technique is the best.

The objective of this study was to compare the effects of conventional suture, polyglactin 910 mesh, and omental flap coverage on healing and anastomotic leak in experimental colonic anastomosis in rats.

## METHODS

### Ethics Approval

The study was approved by the Kafkas University Animal Experiments Local Ethics Committee with the study code (approval nº: KAU-HADYEK/2020/111).

### Animals

The study included 18 Wistar *(Rattus norvegicus albinus)* rats weighing between 250 and 300 g, without considering sex differences. Rats were placed in separate cages under standard laboratory conditions (12 h dark/12 h daylight, 45-55% humidity, and 20-22°C room temperature). Animals were fed ad libitum with standard feed and water.

### Study groups

Animals were divided into three groups. The groups according to the methods of colonic anastomosis are presented as follows:

Group 1(n=6): Primary suture group

Group 2 (n=6): Primary suture *plus* polyglactin 910 mesh group

Group 3 (n=6): Primary suture *plus* omental flap coverage group

### Absorbable Surgical Barrier Film

VICRYL^®^ (polyglactin 910) Woven Mesh & VICRYL^®^ (polyglactin 910) Knitted Mesh was used as absorbable surgical barrier film.

### Anesthesia

The animals were put under general anesthesia by intraperitoneally (IP) injecting a mixture of 10 mg/kg xylazine HCl (Rompun, 2%, Bayer) and 100 mg/kg ketamine HCl (Ketalar, 50 mg/mL, Pfizer).

### Surgery

**Figure 1 -:**
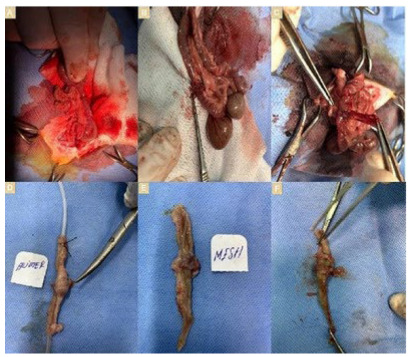
Perioperative anastomosis images: (A) Group 1: Primary suturation group, (B) Group 2: Primary suturation + polyglactin 910 mesh group, (C) Group 3: Primary suturation + omental flap coverage group. Postoperative anastomosis line: (D) Group 1: Primary suturation group, (E) Group 2: Primary suturation + polyglactin 910 mesh group, (F) Group 3: Primary suturation + omental flap coverage group.

Following preoperative 4-h fasting and anesthesia, the abdominal area was shaved and disinfected (povidone iodine + 70% ethanol). After the area was opened by routine methods, the column was accessed, and colon resection (complete layered) was performed in the right colon (ascending colon) in all groups. Colonic anastomosis was conducted by performing primary suture in Group 1 (Figure 1A), primary suture *plus* polyglactin 910 mesh in Group 2 (Figure 1B), and primary suture *plus* omental flap coverage in Group 3 (Figure 1C).

Gambee suture technique and 4/0 polyglactin 910 absorbable suture material (Vicryl) were used as anastomotic suturing material in all groups. The operation was completed by closing the area using routine methods.

### Postoperative Care

All rats were housed in standard cages under standard laboratory conditions, and feed and water were regularly provided to the rats for 7 days.

### Macroscopic Examination

On study day 7, a high dose of pentobarbital sodium was administered by IP route, and euthanasia was performed. Following the euthanasia procedure, the anastomotic colon was accessed after the area was opened using routine methods. The anastomosis line was evaluated macroscopically, and its images were captured. The colon was resected to include the anastomosis line approximately 4 cm proximal and distal to the anastomosis line (Figure 1D-F). Tissue bursting pressure test was performed on the anastomosis line sections, and then these pieces were delivered to the histology laboratory for evaluation in 10% formaldehyde solution.

### Measurement of Anastomosis Bursting Pressure

**Figure 2 -:**
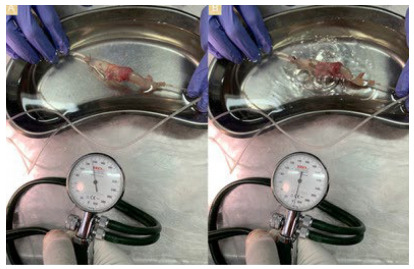
Anastomotic burst pressure measurement. (A) Anastomotic pressure measurement, (B) The moment of the anastomosis burst.

The distal ends of all resected anastomotic colon segments were tightly tied using 2/0 silk sutures. A polyethylene catheter was inserted into the lumen from the proximal end with the other end of the catheter connected to a transducer and an air pump. The necessary setting was thus achieved to display the intraluminal pressure in millimeters of mercury (mmHg). The anastomotic colonic segment was placed in a container filled with water, and air was blown into the lumen at a rate of 2 mL/min. The first air outlet from the anastomotic line was recorded as the anastomotic bursting pressure (Figure 2A, B). The measured bursting pressure values were evaluated using the Kruskal-Wallis analysis of variance test to detect significant statistical differences between groups.

### Histopathological Examination

At the end of the study, tissue samples including the anastomosis region were collected from all groups for histological examination. After the collected tissue samples were fixed in 10% formaldehyde solution, a routine histological tissue follow-up procedure was performed. The tissues were then blocked in paraffin. Then, 5-μm thick sections were taken from the paraffin blocks. Tissue sections were stained using Crossman Modified Triple staining technique for the histological evaluation of the tissues. Tissues were evaluated histologically, and their images were captured. In the histological evaluation of the tissues, scoring between 1 and 4 was made considering inflammatory cells, fibroblastic activity, neovascularization, and the amount of collagen based on the Erlich-Hunt model (1: low and local, 2: low and extensive, 3: dense and local, and 4: dense and extensive).

### Statistical Analysis

The normal distribution of the data within the groups was determined by the Kruskal-Wallis test. Mann-Whitney U test was used for comparison of the groups.

## RESULTS

### Clinical Observations

During the study, all animals maintained their normal lives, and no adverse conditions were present regarding the animals or the anastomosis line. On postoperative day 7, it was macroscopically observed that the recovery in the anastomosis line was smooth in all groups.

### Macroscopic Findings

There were no macroscopic signs of leak, infection, or necrosis in the anastomosis line in all groups.

*Anastomotic Pressure Results:*

The mean anastomotic pressure was 121.67±1.585 in Group 1, 155.33±6.844 in Group 2, and 151.67±4.364 mmHg in Group 3. Based on the comparison of the mean anastomotic pressures between the groups, there was a statistically significant difference between Groups 1 and 2 and between Groups 1 and 3 (p=0.004). However, there was no significant difference between Groups 2 and 3 (p=0.748) (Table 1).

**Table 1 -:** The average anastomotic bursting pressure.

Groups	Bursting pressure (mmHg) X±SE
Group 1	121.67±1.585 ^a^
Group 2	155.33±6.844 ^b^
Group 3	151.67±4.364 ^b^

There is a statistical difference between the groups shown with different letters in the same column (p<0.05).

### Histopathological Findings

**Figure 3 -:**
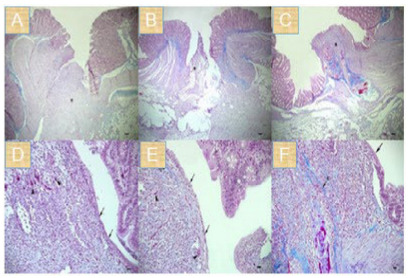
General views of the anastomosis line histopathologically. (A) Group 1: Primary suturation group, (B) Group 2: Primary suturation + polyglactin 910 mesh group, (C) Group 3: Primary suturation + omental flap coverage group. * Anastomosis line. (Crossman Modified Triple staining technique. Bar: 500 μm). (D) Group 1; arrows: re-epithelization, arrowheads: neovascularization, (E) Group 2; arrows: re-epithelization, arrowheads: neovascularization, C: collagen synthesis, (F) Group 3; arrows: re-epithelization, C: collagen synthesis. (Crossman Modified Triple staining technique. Bar: 100 μm).

Based on the histological evaluations, all groups had mucosal and submucosal bridging in the anastomosis line (Figure 3A-C).

*Group 1 (Primary suture)*

Lymphocytes and macrophages were generally found in the anastomosis line in the primary suturation group. Fewer granulocytes were also found. In addition to these cells, connective tissue cells and smooth muscle cells were also found. Furthermore, it was observed that this group had mild fibroblastic activity and a small amount of collagenization. It was determined that there was prominent neovascularization in the tissues as well as incomplete re-epithelization (Figure 3D).

*Group 2 (Primary suture* plus *polyglactin 910 mesh)*

It was determined that in the polyglactin 910 mesh group, the quantity of lymphocyte, monocyte, and macrophage cells in the anastomosis line was normal; the quantity of granulocytes was low; and there were also smooth muscle cells and connective tissue cells. In addition, it was found that there was moderate fibroblastic activity and collagenization in the polyglactin 910 mesh group. It was determined that there was re-epithelization and neovascularization in places (Figure 3E).

*Group 3 (Primary suture* plus *omental flap coverage)*

In the omental flap coverage group, lymphocytes and macrophages were generally found in the anastomosis area, while a small number of granulocytes were also found. Again, as in the other groups, it was noticed that smooth muscle cells and connective tissue cells were present in the anastomosis area in the omental flap coverage group, but the rate of fibroblastic activity and collagenization was higher compared to other groups. It was determined that there was re-epithelization in some areas (Figure 3F).

The histological examinations performed on tissue samples in all groups, and it was determined that the healing in the anastomosis area was in the proliferation phase. Histological scoring based on the Erlich-Hunt model is shown in Table 2.

**Table 2 -:** Scores of the groups based on Ehrlich-Hunt model.

Groups	Inflammation	Fibroblastic activity	Neovascularization	Collagen amount	Total
Group 1	2	1	2	1	6
	2	1	1	1	5
	2	2	1	1	6
	1	1	2	1	5
	1	2	1	2	6
	1	1	2	1	5
Group 2	1	1	2	1	5
	2	1	3	3	9
	2	2	2	2	8
	1	2	2	2	7
	1	2	3	2	8
	2	2	2	3	9
Group 3	1	2	3	2	8
	1	3	3	3	10
	2	2	2	2	9
	1	3	3	3	10
	2	2	2	2	8
	1	3	3	4	11

### Histopathological Statistical Results

Statistical values of the histopathological analysis are presented in Table 3. There was no statistically significant difference in inflammation between the groups (p>0.05).

**Table 3 -:** The average values obtained from the histopathological evaluation of the groups according to Ehrlich-Hunt model and p-values obtained from their statistical comparison.

Groups	Inflammation (X±SE)	Fibroblastic activity (X±SE)	Neovascularization (X±SE)	Collagen amount (X±SE)
Group 1	1.50±0.224	1.33±0.211 ^a^	1.50±0.224 ^a^	1.17±0.167 ^a^
Group 2	1.50±0.224	1.67±0.211 ^a^	2.33±0.211 ^b^	2.17±0.307 ^b^
Group 3	1.33±0.211	2.50±0.224 ^b^	2.67±0.211 ^b^	2.67±0.333 ^b^

There is a statistical difference between the groups shown with different letters in the same column (p<0.05).

There was no statistically significant difference fibroblastic activity between Groups 1 and 2 (p=0.269, p>0.05). There was a significant difference between Groups 1 and 3 (p=0.011, p>0.05) and between Groups 2 and 3 (p=0.030, p<0.05). While Group 3 had the highest fibroblastic activity, Group 1 had the lowest fibroblastic activity.

There as a statistically significant difference in neovascularization between Groups 1 and 2 (p=0.030, p>0.05) and between Groups 1 and 3 (p=0.011, p>0.05), but there was no significant difference between Groups 2 and 3 (p=0.269, p>0.05). The mean neovascularization values were higher in the Groups 2 and 3 compared to Group 1.

There was a statistically significant difference in collagen between Groups 1 and 2 (p=0.023, p<0.05) and between Groups 1 and 3 (p=0.005, p<0.05). However, there was no significant difference between Groups 2 and 3 (p=0.337, p>0.05). The mean collagen amount was higher in Groups 2 and 3 compared to Group 1.

## DISCUSSION

Anastomotic leak continues to cause serious morbidity and mortality in patients undergoing colorectal surgery, so it is difficult to treat and may require re-laparotomy^5^ ^,^ ^19^. Numerous experimental and clinical studies have been conducted to highlight new treatment strategies to prevent anastomotic leak and to achieve better wound healing^1^ ^,^ ^10^ ^,^ ^19^ ^,^ ^23^. The purpose of our study was to demonstrate the effect of an absorbable surgical barrier film on the reliability of the anastomosis by fixing the omentum and the rarely used absorbable barrier film around the anastomosis.

Omentum is a large adipose tissue layer located on the surface of IP organs and has important biological roles in the regulation of immunity and tissue regeneration as well as fat storage^7^ ^,^ ^18^. The omentoplasty technique is used in gastrointestinal surgery to wrap the anastomosis areas, to support the fusion, and to prevent anastomotic leak. A study conducted on 705 patients who underwent bowel resection and anastomosis compared groups which underwent omentoplasty and not omentoplasty and found no difference in anastomotic leak (4.7% vs. 5.2%) and mortality (4.9% vs. 4.2%) between the groups^14^. On the contrary, in a study conducted by Tocchi et al on 112 patients, 3.8% of the patients who underwent omentoplasty developed anastomotic leak, while 11.2% of those who did not undergo omentoplasty developed anastomotic leak; this study suggests that omentoplasty decreases anastomotic leak^22^. In our experimental study on rats, there was a statistically significant difference in anastomotic pressure between Group 1, primary suturation group, and Group 3, suture plus omentoplasty group. Mean anastomotic bursting pressure was 121.67±1.585 mmHg in Group 1 and 151.67±4.364 mmHg in Group 3. It can be concluded that omentoplasty increases the strength of the anastomosis.

In clinical practice, the physical strength of the anastomosis is not an ideal parameter for the evaluation of colonic anastomosis healing;^21^ nevertheless, burst pressure was used as an indirect method in our study in order to evaluate anastomotic integrity. In a study which compared the efficacy of a hemostatic agent on the anastomosis, the mean burst pressure was 193±28.75 mmHg on day 7 in the group that received hemostatic agent and 165±53.45 mmHg in the group that did not receive hemostatic agent^8^. However, the effectiveness of a surgical absorbable barrier film on the anastomotic pressure was compared, and on day 7, the anastomotic pressure measurement results were 190.0±25.82 mmHg in the group that used a barrier film and 146.0±15.06 mmHg in the group that did not use a barrier film^15^. In our study, the mean bursting pressure was 155.33±6.844 mmHg on day 7 in Group 2 in which an absorbable surgical barrier film was used, while it was 151.67±4.364 mmHg in Group 2 in which omentoplasty was performed. However, it was 121.67±1.585 mmHg in Group 1 in which only primary suturation was used. In our study, there was a statistically significant difference between Groups 1 and 2, while there was no significant difference between Groups 2 and 3. It can be concluded that surgical barrier film increases the physical strength of the anastomosis as much as omentoplasty.

Wound healing develops as a result of a series of events consisting of hemostasis and inflammation, proliferation (proliferation of cells), and restructuring and maturation phases in order to restore the integrity and functional capacity of the tissue. Long duration or interruption of any of these phases causes delay in wound healing or the wound to become chronic^12^ ^,^ ^16^. In a study which compared groups which underwent primary suturation and were scored based on the Ehrlich-Hunt model in terms of anastomosis healing, there was no difference in inflammation, neovascularization, fibroblastic activity, and collagen between the groups^15^. Based on the histopathological evaluation, there was no significant difference in inflammatory cells between the groups.

Based on evaluation in terms of fibroblastic activity, in experimental studies which used self-gripping mesh in colonic anastomosis and compared the efficiency of expanded polytetrafluoroethylene patch in duodenal injuries, fibroblastic activity was significantly higher in study groups^2^ ^,^ ^4^. In a study which compared the efficacy of Poly-e-caprolactone scaffold on anastomosis, there was no significant difference in terms of fibroblast activity^13^. Similarly, in another study, Ankaferd Blood Stopper was used on the colonic anastomosis, and there was no difference in terms of fibroblast activity^18^. The omentum has different roles in supporting tissue regeneration^5^. In our study, the highest fibroblastic activity was in the omental flap coverage group, the second highest fibroblastic activity was in the polyglactin 910 mesh group, and the lowest fibroblastic activity was in the primary suture group.

Based on evaluation in terms of neovascularization and collagen, although there was no significant difference in the study groups compared to the control groups in some studies^8^ ^,^ ^13^ ^,^ ^20^, there are studies which showed that neovascularization and collagen were higher in the study groups, as in our study^2^ ^,^ ^4^.

## CONCLUSION

This experimental study found that polyglactin 910 mesh and omental flap coverage for colocolic anastomoses increased the physical strength and healing of the anastomosis compared to conventional hand-stitched anastomoses. It was concluded that polyglactin may be a safe alternative to 910 mesh in cases where the omental flap coverage cannot be used in the colonic anastomosis.
